# Feasibility of Digital Cognitive Behavioral Therapy for Depressed Older Adults With the Moodbuster Platform: Protocol for 2 Pilot Feasibility Studies

**DOI:** 10.2196/41445

**Published:** 2022-10-25

**Authors:** Khadicha Amarti, Mieke H J Schulte, Annet Kleiboer, Claire Rosalie Van Genugten, Mardien Oudega, Caroline Sonnenberg, Gonçalo C Gonçalves, Artur Rocha, Heleen Riper

**Affiliations:** 1 Clinical Psychology Section Department of Clinical, Neuro- and Developmental Psychology Vrije Universiteit Amsterdam Netherlands; 2 Amsterdam Public Health Research Institute Vrije Universiteit Amsterdam Netherlands; 3 Amsterdam Universitair Medisch Centrum Vrije Universiteit Public Health Research Institute and Neuroscience Amsterdam Amsterdam Netherlands; 4 Department of Old Age Psychiatry Geestelijk Gezondheidszorg inGeest Specialized Mental Health Care Amsterdam Netherlands; 5 Institute for Systems and Computer Engineering, Technology and Science Porto Portugal; 6 Institute of Telepsychiatry University of Southern Denmark Odense Denmark; 7 Faculty of Medicine University of Turku Turku Finland

**Keywords:** iCBT, study protocol, feasibility study, acceptance, satisfaction, usability, engagement, depression severity, older adults

## Abstract

**Background:**

Internet-based interventions can be effective in the treatment of depression. However, internet-based interventions for older adults with depression are scarce, and little is known about their feasibility and effectiveness.

**Objective:**

To present the design of 2 studies aiming to assess the feasibility of internet-based cognitive behavioral treatment for older adults with depression. We will assess the feasibility of an online, guided version of the Moodbuster platform among depressed older adults from the general population as well as the feasibility of a blended format (combining integrated face-to-face sessions and internet-based modules) in a specialized mental health care outpatient clinic.

**Methods:**

A single-group, pretest-posttest design will be applied in both settings. The primary outcome of the studies will be feasibility in terms of (1) acceptance and satisfaction (measured with the Client Satisfaction Questionnaire-8), (2) usability (measured with the System Usability Scale), and (3) engagement (measured with the Twente Engagement with eHealth Technologies Scale). Secondary outcomes include (1) the severity of depressive symptoms (measured with the 8-item Patient Health Questionnaire depression scale), (2) participant and therapist experience with the digital technology (measured with qualitative interviews), (3) the working alliance between patients and practitioners (from both perspectives; measured with the Working Alliance Inventory–Short Revised questionnaire), (4) the technical alliance between patients and the platform (measured with the Working Alliance Inventory for Online Interventions–Short Form questionnaire), and (5) uptake, in terms of attempted and completed modules. A total of 30 older adults with mild to moderate depressive symptoms (Geriatric Depression Scale 15 score between 5 and 11) will be recruited from the general population. A total of 15 older adults with moderate to severe depressive symptoms (Geriatric Depression Scale 15 score between 8 and 15) will be recruited from a specialized mental health care outpatient clinic. A mixed methods approach combining quantitative and qualitative analyses will be adopted. Both the primary and secondary outcomes will be further explored with individual semistructured interviews and synthesized descriptively. Descriptive statistics (reported as means and SDs) will be used to examine the primary and secondary outcome measures. Within-group depression severity will be analyzed using a 2-tailed, paired-sample *t* test to investigate differences between time points. The interviews will be recorded and analyzed using thematic analysis.

**Results:**

The studies were funded in October 2019. Recruitment started in September 2022.

**Conclusions:**

The results of these pilot studies will show whether this platform is feasible for use by the older adult population in a blended, guided format in the 2 settings and will represent the first exploration of the size of the effect of Moodbuster in terms of decreased depressive symptoms.

**International Registered Report Identifier (IRRID):**

PRR1-10.2196/41445

## Introduction

### Background

Depression is a common mental disorder that is associated with the substantial loss of a person’s well-being and quality of life [1]. Essential components of depression are depressed mood and loss of interest or pleasure in nearly all activities [2]. Depression constitutes a large and rising proportion of global disease [3] and is a major public health problem [4]. Late-life depression is the third leading contributor to the global burden of disease [5]. Late-life depression can be distinguished from adult depression in several ways. First, depressed older adults may show different or less obvious symptoms than their younger counterparts, and cognitive symptoms (eg, disorientation, memory loss, and distractibility) may be particularly prominent [6]. Cognitive impairment may mask the symptoms of late-life depression, as is often seen in vascular disease, Alzheimer dementia, and Parkinson disease [7]. Second, depression in later life is associated with increased risk of physical and psychological disability, decreased quality of life, and increased costs due to higher health care needs in general [7]. Lastly, depressive symptoms in later life increase the distress of family and friends and the burden of informal caregivers, such as spouses or other family members, which often contributes to social isolation of the older adult with depression [8].

Late-life depression is often not recognized or treated [6]. This is unfortunate, because treatment of late-life depression is effective, and decreases not only depressive symptoms [4], but also secondary symptoms, such as pain, general functioning, and health-related quality of life [9,10]. Depending on the diagnosed type of depression and the severity of the impairment, a variety of treatment interventions can be considered for older adults, such as psychoeducation, psychotherapy, and antidepressants [11]. Although antidepressants are effective as a treatment option for older adults [12], they can put them at greater risk for adverse events compared to younger adults with depression because of multiple medical comorbidities and drug-drug interactions, and can contribute to polypharmacy, which increases with aging [13]. Psychotherapies such as problem-solving therapy, cognitive behavioral therapy (CBT), and interpersonal psychotherapy are associated with fewer risks and have benefits equal to antidepressants [13]. CBT is an especially well studied [13,14] and effective type of psychotherapy for depression in later life [15,16]. Unfortunately, only a small proportion of older adults seek psychological treatment [17]. Internet-based interventions can help bridge this treatment gap [18].

### Internet-Based CBT

In recent years, internet-based interventions for the treatment of various common mental disorders, including depression, have been developed and evaluated [19]. These online treatments may overcome barriers that hinder access to face-to-face treatment, such as costs, long waiting lists, limited access to psychological treatment in particular neighborhoods, and the perceived stigma of seeking treatment for a psychiatric disorder [20]. Internet-based CBT (iCBT) provides the same information and teaches similar skills as traditional face-to-face CBT, but does so through the internet with structured materials [21-23]. Additionally, iCBT interventions can be provided in different ways: with therapeutic guidance (ie, guided), without human support related to the therapeutic content (ie, unguided), or in a blended format (ie, integrating online and face-to-face sessions) [24-26]. Ample research has shown that iCBT interventions for depression are effective compared to inactive and active controls. Guided iCBT outperforms unguided interventions, especially among more severely depressed individuals [19,27,28].

Research into internet-based interventions for older depressed adults is scarce, underlining the need for more research to be able to assess the feasibility and effectiveness of these interventions for this specific group. A study by Spek et al [21] measured the effectiveness of an unguided CBT intervention for older adults (aged between 50 and 60 years) and found that it was at least as effective as face-to-face group CBT in reducing symptoms of depression. Furthermore, a randomized controlled trial (RCT) performed by Titov et al [29] supported the use of therapist-guided iCBT as an evidence-based approach to psychological treatment for older adults (aged between 60 and 76 years) with depression. Despite the fact that older adults are generally positive about internet-based interventions [30], there are hardly any digital interventions for the treatment of depression that specifically target older adults, either within the general population or in specialized mental health care outpatient clinics. Also, with the growing number of older adults in our society, there has been a large rise in the number of patients suffering from depression [31]. Furthermore, older adults are not always able to engage with traditional face-to-face interventions, as they might have limited access to health services due to physical health and constraints or due to limited mobility for other reasons, such as a lack of public transport. The need for innovative and effective digital interventions for older adults is thus a public health priority. Lastly, the study by Spek et al [21] recruited participants from the general population and provided the intervention in an unguided format. We therefore plan to evaluate the feasibility of using the intervention in a specialized mental health care outpatient clinic with a blended format and in the general population with a guided format.

### Aim of the Current Studies

The 2 current studies aim to evaluate the feasibility of an online treatment platform (Moodbuster) that targets depressive symptoms in older adults (aged at least 55 years) in 2 settings. The first study will test the feasibility of Moodbuster with an online guided treatment format for older adults with mild to moderate depressive symptoms recruited from the general population. The second study will test the intervention in a blended treatment format by integrating face-to-face and online sessions into one treatment protocol. It will target older adults with moderate to severe depression recruited from a specialized mental health care outpatient clinic. The primary aim of both studies is to evaluate the feasibility of the online platform in terms of (1) acceptance and satisfaction, (2) usability, and (3) engagement. Secondary outcomes include depressive symptom severity, participant experience with digital technologies, technical alliance, working alliance, and uptake.

## Methods

### Study Design

**Figure 1:**
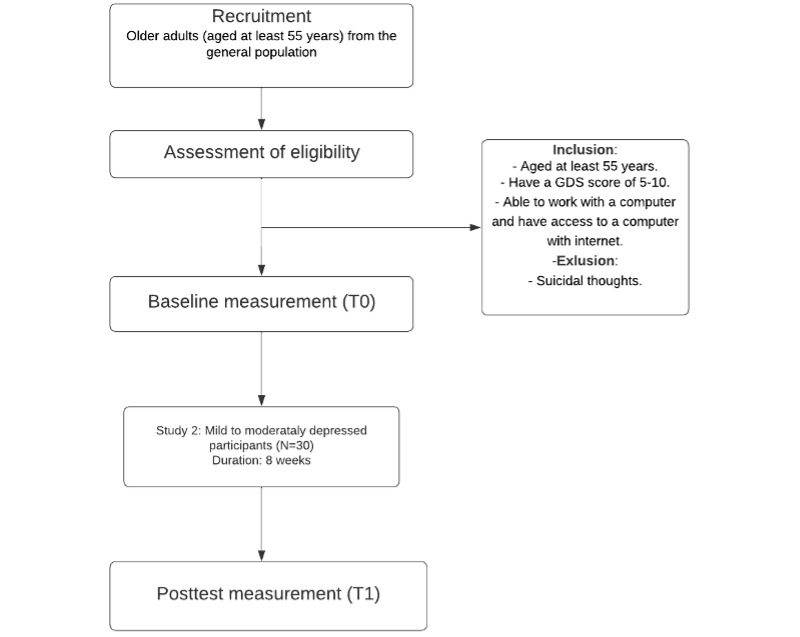
Flowchart of the study design for the group of older adult with mild to moderate depressive symptoms. GDS: Geriatric Depression Scale.

**Figure 2:**
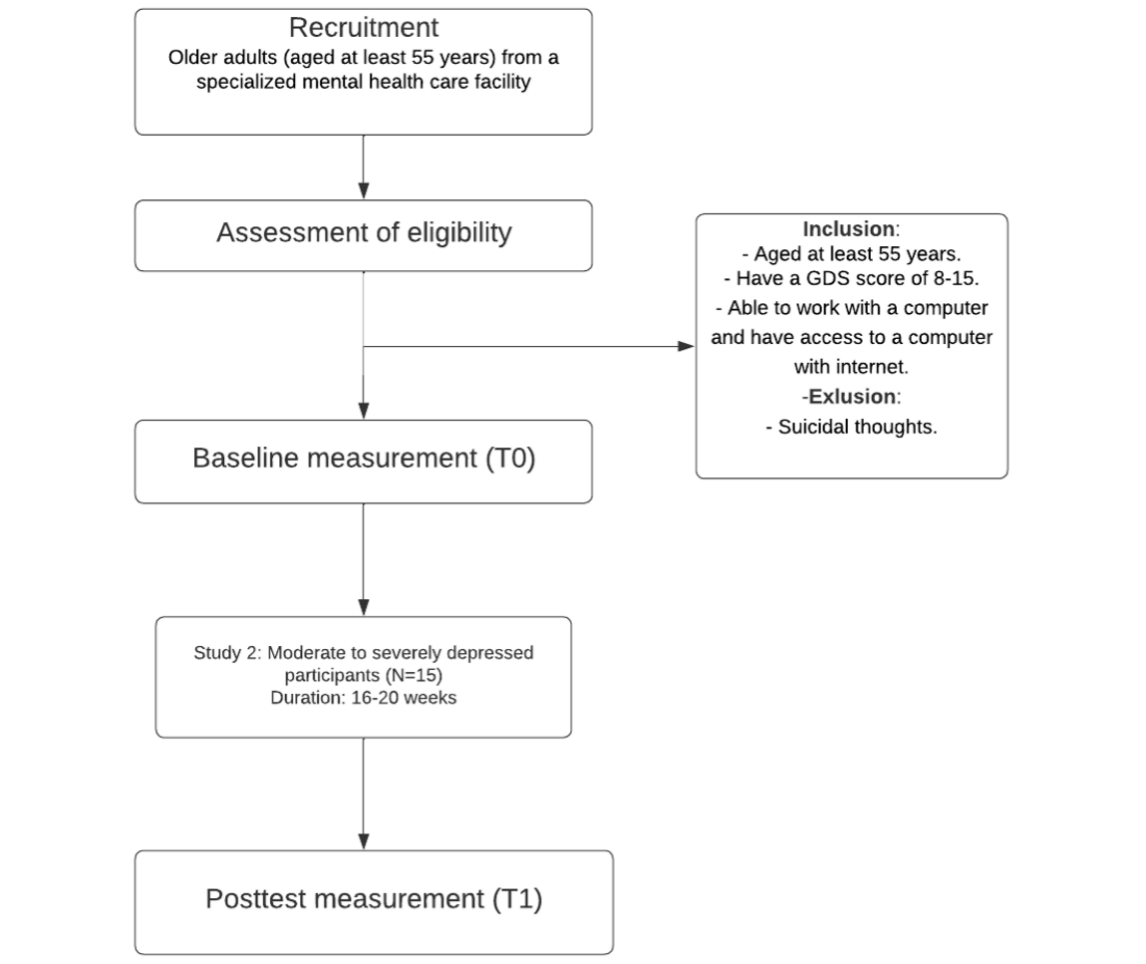
Flowchart of the study design for the group of older adult with moderate to severe depressive symptoms. GDS: Geriatric Depression Scale.

A single-group, pretest-posttest design will be used in both studies. Assessments will be taken at screening (T–1), at baseline (T0), and postintervention (T1). The intervention will last 8 weeks for the group with mild to moderate depressive symptoms from the general population and 16 to 20 weeks for the group with moderate to severe depressive symptoms from the specialized mental health care outpatient clinic. Written informed consent will be obtained from all participants. Figures 1 and 2 show the participant flow for both studies.

### Participants

#### General Population

Participants with mild to moderate depressive symptoms will be recruited from the general population in the Netherlands. They will be eligible to participate if they (1) are aged at least 55 years, (2) have a Geriatric Depression Scale-15 (GDS-15) score between 5 and 10, (3) are able to work with a computer, and (4) have access to a computer with internet. Candidates will be excluded from the study if they (1) do not have adequate proficiency in the Dutch language or (2) have suicidal thoughts, assessed as having a score of 1 or higher on the 9th item of the Patient Health Questionnaire 9 (PHQ-9) [32]. Having suicidal ideation is considered an exclusion criterion to minimize the risk of an adverse event during the study period.

#### Specialized Mental Health Care Service

Participants with moderate to severe depressive symptoms will be recruited via a specialized mental health care outpatient clinic (Geestelijk gezondheidszorg [GGZ] inGeest, Amsterdam, the Netherlands). Patients who are in treatment at the clinic will be eligible to participate if they (1) are aged at least 55 years, (2) have a GDS-15 score between 8 and 15, (3) are able to work with a computer, and (4) have access to a computer with internet. Candidates will be excluded from the study if they (1) do not have adequate proficiency in the Dutch language or (2) have suicidal thoughts, assessed as having a score of 1 or higher on the 9th item of the PHQ-9 [32]. Having suicidal ideation is considered an exclusion criterion to minimize the risk of an adverse event during the study period.

### Study Intervention: Online Treatment Platform

**Figure 3:**
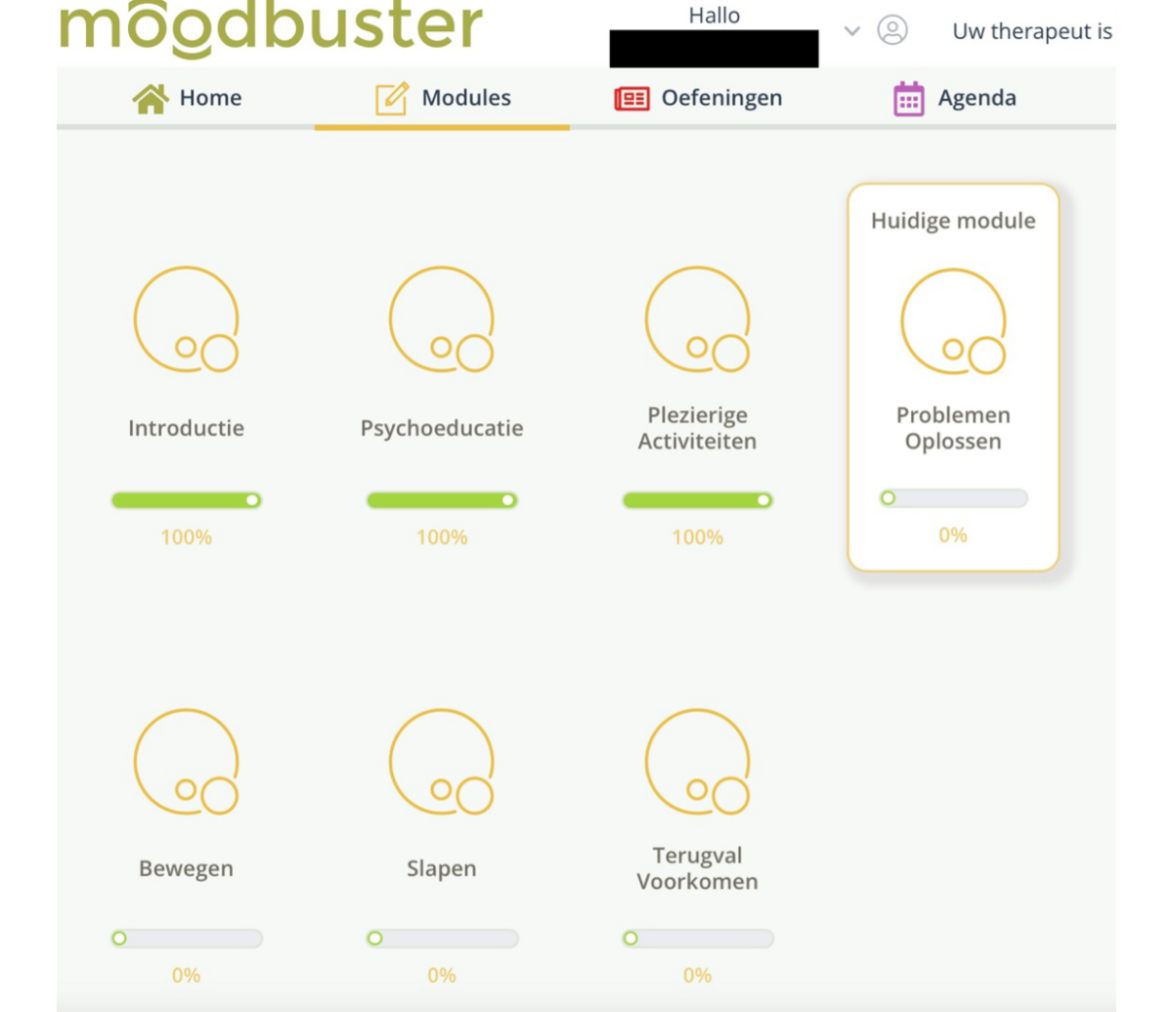
Screenshot of the patient portal of Moodbuster 2.0.

Moodbuster 2.0 has been developed and tested in a number of European projects. It is an innovative online and mobile solution for the treatment of adult depression and can be applied for research purposes. The platform has a patient and therapist portal (Figure 3) and can be used in unguided, guided, or blended formats and in different settings [33-35]. For the purpose of these studies, Moodbuster 2.0 has been adapted to meet the needs of depressed older adults. To make Moodbuster more aligned with the target group of older adults, the original Moodbuster modules were adjusted by making the content shorter and more relevant to older adults.

Moodbuster 2.0 for older adults consists of 7 web-based CBT modules. These modules are aimed at psychoeducation, behavioral activation, cognitive therapy, problem-solving, exercise, sleep, and relapse prevention. The sleep module is a new addition we have made to the platform; it is aimed at older adults, as insomnia and other sleep-related problems are strongly associated with the persistence of depressive symptoms in older adults [1,36-38]. Texts and videos lead the user through the modules, during which exercises are performed and homework assignments are given.

The online guided treatment for older adults from the general population includes the support of an online coach [39]. Participants receive online CBT over a period of 7 to 8 weeks. The participants are instructed to complete 1 module each week. The psychoeducation module is obligatory. We advise the participants to start with the behavioral activation and cognitive therapy modules. The problem-solving, exercise, sleep, and relapse-prevention modules are optional and are used according to the preferences of the participant. Online support for the guide consists of actively giving the participants feedback for the assignments (at the least after every completed module) using the secure messaging system included in the Moodbuster platform. Furthermore, the guide can answer any questions the participants might have.

The blended CBT treatment that will be offered to participants with moderate to severe depressive symptoms is based on evidence-based CBT protocols implemented in routine practice [40]. The blended format provides individuals face-to-face sessions and online sessions. Participants receive treatment over a period of 16 to 20 weeks, depending on the intensity of the treatment. The therapist and patient decide together the best way to organize face-to-face sessions and online sessions. For example, they can schedule an online session after each face-to-face session or an online session after 2 face-to-face sessions. The practitioner should provide feedback for the online sessions within the face-to-face sessions.

### Study Procedure

The participants will be recruited from the 2 target groups and settings simultaneously. Participants with mild to moderate depressive symptoms (as assessed with the GDS-15) [41] will be recruited from the general population via advertisements in digital media and an online recruitment platform, Link2Trials (Link2Trials BV). For this group the following steps will be taken: potential participants can express their interest by filling out a web form, after which they will receive an information letter and a printed informed consent form via postal mail. When the signed informed consent form is received, it will be scanned and safely stored at the department. Participants who sign the consent form will receive a link to the online screening questionnaires (T–1) through a secure electronic data capture (EDC) system and, once found eligible for participation, they will receive a link to the baseline questionnaires (T0) through Castor EDC (Castor Research Inc). Once the baseline questionnaires are completed, another email will be sent with login credentials for accessing the Moodbuster 2.0 platform. Participants are strongly advised to change their password upon the first login. Participants are advised to work through the intervention in 7 to 8 weeks (ie, 1 module per week). After a maximum of 8 weeks of participation, participants will receive another email with a link to another set of questionnaires to acquire posttreatment assessments (T1). After completing the posttreatment assessments, participants will receive an email to thank them for their participation and ask them if they wish to be informed about the study’s results. After filling in the last questionnaire, participants will be reimbursed for their participation with €30 (US $29.41). After the treatment has ended, the participants are invited to fill in an open-field questionnaire to evaluate their experience with Moodbuster.

Patients with moderate to severe depressive symptoms will be recruited from the patient population of the specialized mental health care outpatient clinic. For this group, the following steps will be taken: patients who express interest during intake with their therapist will receive an information letter and an informed consent form at the end of the session. Patients who sign the consent form will be assessed by their therapist to confirm they have moderate to severe depressive symptoms (as assessed with the GDS-15 [41]). Once found eligible for participation, they will receive a link to the baseline questionnaires (T0) through Castor EDC. Once the baseline questionnaires are completed, another email will be sent with the login credentials for accessing the platform. The GGZ inGeest therapists will aid participants with logging in on the platform the first time they use it. Participants are strongly advised to change their password upon the first login. Also in this step, the practitioners will assist the participants with becoming familiar with the platform and answer any questions they might have. It will also be communicated that participants should contact their therapist if they feel their symptoms are suddenly deteriorating. Participants will use the platform in a blended format. The therapists will be trained on how to use the platform before the participants start the treatment. After 16 to 20 weeks of participation (depending on the duration of the treatment), the participants will receive another email with a link to another set of questionnaires to acquire posttreatment assessments (T1). After completing the posttreatment assessments, the participants will receive an email to thank them for their participation and ask them if they wish to be informed of the study’s results. After the treatment ends, 5 participants will be invited to participate in individual interviews, which will be conducted face-to-face, through videoconferencing, or by telephone, to evaluate their experience with Moodbuster.

### Assessments

Questionnaires will be completed online for both groups. Data will be collected and managed using Castor EDC. Table 1 provides an overview of the measures employed at specific time points.

**Table 1:** Measures at each assessment interval.

Questionnaires			Aim		Screening (T-1)		Baseline (T0)		Posttreatment (T1)
**Screening**									
	Geriatric Depression Scale-15	Depressive symptoms		✓					
	Item 9 of Patient Health Questionnaire-9	Suicidal ideation		✓					
**Feasibility**									
	Client Satisfaction Questionnaire-8	Acceptability/ satisfaction						✓	
	System Usability Scale^a^	Usability						✓	
	Twente Engagement with eHealth Platforms Scale	Engagement				✓		✓	
**Secondary outcome measures**									
	Patient Health Questionnaire-8	Depressive symptoms				✓		✓	
	Working Alliance Inventory—Short Form^a^	Working alliance						✓	
	Working Alliance Inventory Technical—Short Form	Technical alliance						✓	
	Uptake	Continued use						✓	
	Digital experiences	General experiences				✓			
	Digital Health Literacy Instrument	Digital literacy				✓			

^a^Measure taken from from participants and practitioners.

### Screening

#### Depression Severity

The GDS-15 [41] will be used as a screener to assess depression severity, with a cutoff between 5 and 10 for the group with mild to moderate depressive symptoms and a cutoff of 11 or higher for the group with moderate to severe depressive symptoms. The GDS-15 has 15 items with the option to respond with yes or no. Total scores range from 0 to 15, with higher scores indicating higher severity of depression. The GDS-15 has been found to be a reliable and valid instrument [42].

#### Suicidal Ideation

Participants will be screened for suicidal ideation using item 9 from the PHQ-9 [32] and excluded. Item 9 of the PHQ-9 asks about thoughts of being better off dead or hurting oneself in some way over the previous 2 weeks. Response options include 0 (“not at all”), 1 (“several days”), 2 (“more than half the days”), or 3 (“nearly every day”). A higher score indicates higher suicidal ideation. Item 9 on the PHQ-9 is a robust predictor of suicide attempts and deaths regardless of age [43].

### Primary Outcome Measures

#### Acceptance and Satisfaction

Acceptance and user satisfaction will be measured with the Client Satisfaction Questionnaire-8 (CSQ-8) for internet-based interventions [44]. The CSQ-8 is composed of eight 4-point Likert-scale items with response options ranging from 1 (“does not apply to me”) to 4 (“applies to me”). Total scores range from 8 to 32, with higher scores indicating higher levels of client satisfaction. Scores between 8 and 13 indicate poor satisfaction, scores between 14 and 19 indicate fair satisfaction, scores between 20 and 25 indicate good satisfaction, and scores between 26 and 32 indicate excellent satisfaction [45]. The CSQ-8 has been found to be a reliable instrument (Cronbach α=.87) [46].

#### Usability

Usability will be measured with the System Usability Scale (SUS), which was developed by Brooke [47], and with logfile analysis. The SUS has ten 5-point Likert-scale items with response options ranging from 0 (“strongly disagree”) to 4 (“strongly agree”). Total scores are converted (by multiplying the total score by 2.5) to a scale ranging from 0 to 100, where higher scores are indicative of higher platform usability. The SUS is considered a reliable instrument (Cronbach α=.90), with scores higher than 70 indicating “good” usability [48,49].

#### Engagement

Engagement with the platform will be measured with the Twente Engagement with eHealth Technologies Scale (TWEETS), developed by Kelder and Kip [50]. The TWEETS is a 9-item self-reported scale that can be used to measure expectations of engagement, current engagement, or past engagement with digital interventions. In the current studies, expected and past engagement will be assessed. The questions are answered on a 5-point Likert scale in which higher scores indicate higher levels of treatment engagement. The total score is derived by summing the scores for each of the 9 items on a 0 to 4 scale and can range from 0 to 40. The TWEETS has shown to be a valid tool that possesses good psychometric qualities to assess engagement with eHealth technologies (Cronbach α=.87) [51].

### Secondary Outcome Measures

#### Depression Severity

Depression severity will be assessed with the Patient Health Questionnaire-8 (PHQ-8) [52]. The PHQ-8 is composed of 8 items with response options ranging from 0 (“not at all”) to 3 (“nearly every day”). Total scores range from 0 to 24, with higher scores indicating higher severity of depression. Scores between 5 and 9 indicate mild depression, scores between 10 and 14 indicate moderate depression, scores between 15 and 19 indicate moderately severe depression, and scores between 20 and 24 indicate severe depression. The PHQ-8 has been found to be a reliable and valid instrument (Cronbach α= 0.82) [52,53].

#### Working Alliance

Working alliance will be measured using the short version of the Working Alliance Inventory–Short Form (WAI-SF) [54] which assesses the therapeutic alliance between the practitioner and participant. The questionnaire covers three dimensions of working alliance: (1) therapeutic goals, (2) tasks, and (3) bond. The WAI-SF for patients has 12 items with responses on a 5-point Likert scale, ranging from 1 (“never or rarely”) to 5 (“very often”). The WAI-SF for therapists has 10 items with responses on a 5-point Likert scale ranging from 1 (“never”) to 5 (“always”). The WAI-SF has been found to be a reliable and valid instrument for both patients and therapists (Cronbach α=.86 and α=.88, respectively) [54,55].

#### Technical Alliance

Technical alliance will be measured with the Dutch Working Alliance Inventory for Online Interventions-Short Form (WAI-TECH-SF) [56]. This questionnaire assesses the therapeutic alliance between participant and online platform. The questionnaire was designed to cover three dimensions: (1) therapeutic goals, (2) tasks, and (3) bond. The WAI-TECH-SF has 12 items with responses rated on a 7-point Likert scale, ranging from 1 (“never”) to 7 (“always”). The total score ranges from 12 to 84, with higher scores indicating a better technical alliance. The WAI-TECH-SF has been found to be a reliable instrument (Cronbach α=.97) [56].

#### Uptake

Uptake refers to the degree to which a participant engages with the content of the intervention by using or not using [57] the intervention, in this case the Moodbuster 2.0 iCBT or blended CBT. This will be measured with a logfile analysis. This analysis measures logins, time spent on the platform, and number of modules attempted and completed [58]. Additionally, for the blended CBT intervention this will be measured with feedback from the therapist on the number of face-to-face sessions attended and the number of messages sent to the participants.

#### Participant Experiences

Participants’ experiences with the platform will also be explored by means of semistructured interviews. This is to gain a better understanding of participants’ satisfaction with the use of Moodbuster. Qualitative interviews allow the participants more freedom in how to respond [59]. These interviews will focus on issues such as personal experiences with the platform, self-management strategies used by the participants, and contacts between participants and practitioners or guides. For the group with mild to moderate depressive symptoms (ie, the guided group) this will be done by means of an open-ended questionnaire after the last session. For the group with moderate to severe depressive symptoms (ie, the blended therapy group) this will be done by means of a number of individual interviews. The interviews will be conducted by telephone, by video conferencing, or be face-to-face, depending on the preferences of the participant.

### Other Measures

#### Demographic Information

Demographic information will include age, sex (as assigned at birth), living situation, educational level, relationship status, daytime activities, and whether a participant has children.

#### Digital Literacy

Digital literacy is operationalized as the degree of a person’s knowledge, comfort, and perceived skills on a digital instrument such as a computer. Digital literacy will be measured using 2 subscales of the Digital Health Literacy Instrument (DHLI), developed by van der Vaart and Drossaert [60]. The 2 subscales of the DHLI that will be used are “operational skills” and “adding content.” The operational skills subscale is composed of 3 items, with response options ranging from 1 (“very easy”) to 4 (“very hard”). Total scores range from 3 to 9, with higher scores indicating a lower degree of operational skills with digital technology. The operational skills subscale has been found to be reliable (Cronbach α=.77) [60]. The adding content subscale is composed of 3 items, with response options ranging from 1 (“very easy”) to 4 (“very hard”). Total scores range from 3 to 9, with higher scores indicating a lower degree of ability to add content on a digital tool. The adding content subscale has been found to be reliable (Cronbach α=.89) [60].

### Sample Size

Calculating the sample size for a pilot study is not standardized. Sample size can range from 15 to 100 participants and depends on the aim of the study [61]. Some studies recommend at least 12 participants [62] and other studies recommend 30 or more participants [63]. The target group from the general population is characterized by variation in both age and the nature of depressive symptoms. We will therefore recruit 30 participants from the general population. We will recruit patients receiving specialized mental health care from a smaller pool (only patients at GGZ inGeest); this population is also characterized by more severe depressive symptoms, and it will require more time to complete recruitment. Given the time frame for recruitment, we will therefore recruit 15 participants.

### Statistical Analysis

When the data collection is completed, the data will be cleaned and assessed for accuracy. The data from participants from the general population will be analyzed separately from the data from the participants that are recruited from the specialized mental health care outpatient clinic.

### Feasibility Parameters

Quantitative analysis will be conducted using SPSS (version 25; IBM Corp). The primary outcomes will only be assessed after the completion of the interventions (T1). Descriptive statistics (reported as means and SDs) will be calculated to examine and summarize the acceptance and satisfaction of the participants, system usability, and participant engagement with the platform.

### Secondary and Other Study Parameters

Descriptive statistics will be also used to assess the working alliance, technical alliance, and the participants’ use of the intervention (ie, uptake). To investigate baseline differences between participants who complete the studies and those who drop out, a 2-tailed, independent-sample *t* test or a Mann-Whitney *U* test will be used. The within-group secondary outcome of depressive severity at posttreatment will be inspected for outliers and a normal distribution. For each group, the change in depression severity pre- and postintervention will be analyzed using a 2-tailed, paired-sample *t* test with a significance level of α<.05. Effect size will be measured using Hedges *g*, and will be interpreted as follows: small (0.2), medium (0.5), and high (0.8) [64].

### Qualitative Interviews

The interviews and questionnaires will be analyzed using thematic analysis [65]. Thematic analysis is an accessible and flexible method of qualitative data analysis. It identifies and categorizes themes captured in a study. These themes are then coded and organized into meaningful themes. Lastly, interpretative analyses are conducted.

### Data Management

All raw data will be collected and managed using Castor EDC. Paper-based documents (such as signed informed consent forms) will be stored in a keycard-secured archive at the Department of Clinical, Neuro- and Developmental Psychology, Vrije Universiteit Amsterdam. All participants will receive a random study participant code. In the studies, participants will be referred to exclusively by that participant code, and the document linking the numbers will be destroyed once the studies are completed and the results have been disseminated. FAIR (Findability, Accessibility, Interoperability and Reusability) principles of data management will be applied. After data collection is completed, data will be kept in a repository (DarkStor) that serves as an offline archive for storing sensitive information and data.

### Ethics Approval

Ethics approval was granted by the University Medical Centre, Amsterdam (2021.0435).

## Results

Study enrollment started in September 2022. The studies were funded in October 2019 by ZorgOnderzoek Nederland–Medische Wetenschappen (ZonMw). Few studies have studied the acceptability, satisfaction, usability, and engagement of iCBT in a guided and blended format for older adults. In our pilot studies, we expect that iCBT in a guided and blended format will prove acceptable and usable by older adults with depressive symptoms. Furthermore, we expect that symptoms of depression will decrease after following the online (guided or blended) treatment. According to the CONSORT (Consolidated Standards of Reporting Trials) guidelines [66] for reporting of feasibility studies, each objective result will be outlined, including expressions of uncertainty and estimations. Furthermore, the results of any other analysis we perform that could be of use for a future definitive trial will be reported. The manuscript will be submitted to the appropriate journals for dissemination after the final data are analyzed.

## Discussion

### Principal Findings

The results of these pilot studies will show whether Moodbuster, an online depression treatment platform, is feasible for use by older adults in 2 study settings. We will measure (1) acceptance and satisfaction with the platform, (2) usability of the platform, and (3) engagement with the platform. The treatment will be evaluated in 2 target groups with 2 formats: (1) participants with mild to moderate depressive symptoms in the general population, who will use a guided format, and (2) participants with moderate to severe depressive symptoms at a specialized mental health care outpatient clinic, who will use a blended format. In these pilot studies, a possible main finding could be that older adults will find that online treatment and the use of technology are acceptable. Furthermore, we expect that the platform will be usable by the participants, as well as by the therapists and guides. Lastly, we expect that the participants will be engaged with the platform. iCBT interventions have been widely studied and have been proven effective for a variety of common mental health disorders, including depression [27,28]. Little is yet known as to whether older adults are more or less likely to benefit from internet-based interventions compared to their younger counterparts, as there are very few interventions and RCTs specifically aimed at studying iCBT interventions for older adults. This underlines the need for more research to be able to assess the feasibility and effectiveness of these interventions for this specific group.

### Strengths and Limitations

This pilot study is a first step in the development and evaluation of a digital intervention for older adults. This approach avoids wasting resources and placing unnecessary burdens on participants, because feasibility issues will be identified and addressed prior to the main RCT. Furthermore, this study will be the first empirical study of older adults in 2 settings and in 2 different groups. We have assessed the power of our sample size according to the recommendations and guidelines for conducting pilot studies found in the existing literature. However, it might prove to be the case that our sample size is too limited, which might influence the results we obtain.

### Future Directions

Given the limited number of studies on this topic, more research is needed to demonstrate the possibilities of online mental health care for older adults with depression in unguided, guided, and blended settings. The results of this study may provide valuable information on next steps, such as an RCT for testing the clinical effectiveness and cost-effectiveness of iCBT for depressed older adults, and may potentially lead the way to its future implementation in routine care.

## Abbreviations

CBT: cognitive behavioral therapy
CSQ-8: Client Satisfaction Questionnaire-8
DHLI: Digital Health Literacy Inventory
EDC: electronic data capture
GDS: Geriatric Depression Scale
GGZ inGeest: Geestelijk Gezondheidszorg inGeest
iCBT: internet cognitive behavioral therapy
PHQ-8: Patient Health Questionnaire-8
PHQ-9: Patient Health Questionnaire-9
RCT: randomized controlled trial
SUS: System Usability Scale
TWEETS: Twente Engagement with eHealth Technologies Scale
WAI-SF: Working Alliance Inventory–Short Form
WAI-TECH-SF: Working Alliance Inventory for Online Interventions–Short Form
ZonMw: ZorgOnderzoek Nederland–Medische Wetenschappen

## Data Availability

The data sets generated during and/or analyzed during the current study are available from the corresponding author on reasonable request.
